# Cognitive dysfunction and health-related quality of life among older Chinese

**DOI:** 10.1038/srep17301

**Published:** 2015-11-25

**Authors:** Chen-Wei Pan, Xingzhi Wang, Qinghua Ma, Hong-Peng Sun, Yong Xu, Pei Wang

**Affiliations:** 1 Jiangsu Key Laboratory of Preventive and Translational Medicine for Geriatric Diseases, School of Public Health, Medical College of Soochow University, Suzhou, China; 2 Saw Swee Hock School of Public Health, National University of Singapore, Singapore; 3 The 3rd People’s Hospital of Xiangcheng District, Suzhou, China

## Abstract

We aimed to assess the association of cognitive dysfunction with health-related quality of life (HRQOL) among older adults in China. We analyzed community-based cross-sectional data of 5,557 Chinese individuals aged 60 years and above in the Weitang Geriatric Diseases Study. Cognitive dysfunction and HRQOL were assessed using the Abbreviated Mental Test (AMT) and the European Quality of Life-5 dimensions (EQ-5D), respectively. We estimated the impacts of cognitive dysfunction on the EQ-5D index and visual analogue scale (VAS) scores using linear regression models, and the association between cognitive dysfunction and self-reported EQ-5D health problems using logistic regression models. The EQ-5D index and VAS scores were significantly lower for individuals with cognitive dysfunction than their counterparts. After controlling for covariates, the differences in EQ-5D index and VAS scores between individuals with and without cognitive dysfunction were −0.016 (95% confidence interval [CI]: −0.024, −0.008), and −3.4 (95% CI: −4.5, −2.4), respectively. Cognitive dysfunction was associated with reporting of problems in pain/discomfort (odds ration [OR]: 1.37; 95% CI: 1.12, 1.69), and anxiety/depression (OR: 2.13; 95% CI: 1.41, 3.23). The negative impact on HRQOL increased with the severity of cognitive dysfunction. The results indicate cognitive dysfunction was associated with worse HRQOL in older adults.

In China, rapid changes of age structure, characterizing as low fertility rates and decreased mortality rates, occurred during the past few decades. As a result, age-related disorders such as cognitive dysfunction have imposed more and more disease and social burden. Cognitive dysfunction is an umbrella term describing any characteristic impeding the cognition process, ranging in severity from mild cognitive impairment^1^,^2^ to dementia^2^. In two major studies, the prevalence of dementia and cognitive impairment without dementia among older adults aged 60 years and above are 2.8%^3^ and 12.7%^4^, respectively.

Deficits in cognitive dimensions such as memory, attention, orientation, language, and executive function may negatively affect people’s life on various aspects. Impaired verbal abilities may lead to communication difficulties which hinder a person’s ability to maintain social roles at desirable levels^5^; attention deficits may result in physical impairments, self-reported disability^6^ and poor functioning of activities in daily living such as eating, bathing, and personal hygiene^7^; deficits in attention, memory, and executive function may be linked with the mechanisms of pain chronicity^8^; awareness of cognitive dysfunction may cause depression^9^.

Given the complexity of influence of cognitive dysfunction, a comprehensive measure of patient-reported outcomes such as health-related quality of life (HRQOL) is needed. Although the definitions of HRQOL vary slightly^10^,^11^,^12^, they share some common elements including multi-dimensionality (e.g. physical, emotional, and social functioning), subjectivity, and self-assessment^13^. As stated above, HRQOL dimensions may be influenced by cognitive dysfunction. However, if persons with cognitive dysfunction do not view or aware their functioning as being impaired, they may still rate their HRQOL as being not deteriorated. Indeed, two review papers had inconsistent findings: Mitchell *et al*. suggested that cognitive impairment may affect quality of life dimensions in patients with neurological disease^14^; while Banerjee *et al*. indicated that there is no convincing evidence of association between cognitive impairment and HRQOL in patients with dementia^15^. Empirical studies in both non- and institutionalized older populations have also shown that the association is unlikely to be straightforward. For non-institutionalized populations, Johansson *et al*. reported that cognitive dysfunction, even mild impairment, was significantly related to lower HRQOL in 85-year-olds in Sweden^16^; Missotten *et al*. (2008) found that only dementia but not mild cognitive impairment was associated with worse quality of life in older Belgian people^17^; Davis *et al*. suggested that cognitive function was not associated with HRQOL in adults aged over 70 years in Canada^18^. For institutionalized populations, Damián *et al*. reported that worse HRQOL was not related to impaired cognitive function in nursing home (NH) residents in Spain^19^, while Chouiter *et al*. found that HRQOL dimensions were associated with cognitive impairment in Swiss NH residents, though the associations were not all linear^20^.

Apart from the subjective nature of HRQOL, the inconsistent findings may also be due to difference in sample size, statistical method, and inclusion criteria. Using small or moderate sample size^16^,^17^,^18^,^19^ may restrict statistical power to detect the association; not using multivariate regression models controlling for possible confounders^16^,^17^,^18^ may either mask or exaggerate the true effect; applying different inclusion criteria may also influence the relationship (e.g. including or excluding dementia patients).

In this study, we aimed to investigate the relationship between cognitive dysfunction and HRQOL in a large Chinese population of noninstitutionalized older adults using multiple regression models.

## Methods

### Study population

The Weitang Geriatric Diseases Study is a community-based survey conducted in the Weitang town located in Suzhou, an urban metropolis in East China. The aim of the study was to estimate the patterns, predictors and burden of common health conditions of community-dwelling older people aged 60 years and above in East China. Based on the official records, there are 6,030 individuals aged 60 years and above in the town. At the recruitment stage, an invitation letter with the study objectives explained was sent to each family, inviting all the adults aged 60 years and above to participate in the study. The exclusion criteria were: 1) younger than 60 years old; 2) migration from the residing address; 3) living period less than 6 months in the Weitang town; and 4) death. From August 2014 to February 2015, a total of 4,611 subjects (82.1%) attended the clinic. 1,002 subjects did not attend the clinic and were interviewed at home. Therefore, 5,613 individuals were included in the study and 5,557 individuals were interviewed regarding the information on HRQOL. The Weitang Geriatric Diseases Study was conducted following the tenets of the Helsinki Declaration and approved by the Institutional Review Board of Soochow University. All participants gave written informed consent at the recruitment stage of the study.

### Cognitive dysfunction measure

Cognitive dysfunction was assessed using the Abbreviated Mental Test (AMT), which is a commonly used tool for measuring cognitive function^21^. The AMT uses a 10-item scale: age, time of day, year, place, recognition of people, date of birth, national day, president, counting backward from 20 to 1, and recall of an address. Each scale is with a score and the maximum score is 10. A score between 0 to 3, 4 to 7, and 8 and above suggests severe impaired function, moderate impaired function, and normal cognitive function, respectively^22^. To improve relevance to the local context, we made changes to some culturally-specific items. For example, we asked respondents when the national day of China is. The AMT has been validated against the Mini-Mental State Examination in Chinese older populations^23^,^24^.

### Health-related quality of life measure

The European Quality of Life-5 Dimensions questionnaire (EQ-5D), a widely used generic HRQOL instrument, was adopted in the study^25^. The EQ-5D assesses a respondent’s health status on the day of survey in five dimensions: mobility, self-care, usual activities, pain/discomfort, and anxiety/depression. Each dimension has three response alternatives: no problems, moderate problems, and extreme problems. The responses to the five dimensions can be translated into a single preference-based summary score (i.e. EQ-5D index score), anchored on an interval where 0 and 1 represent death and full health, respectively^26^. The EQ-5D also includes a visual analogue scale (VAS) recording a respondent’s self-rated overall health on a vertical scale ranging from 0 (worst imaginable health state) to 100 (best imaginable health state). The EQ-5D has demonstrated validity and responsiveness in older persons with cognitive dysfunction^27^ and showed satisfactory validity and reliability in mainland China^28^,^29^,^30^. We hypothesized that people with cognitive dysfunction would be more likely to report EQ-5D problems in all dimensions.

### Assessment of covariates

The survey also collected information about participants’ socio-demographic characteristics (i.e. age, gender, education level, working status, marriage status, dwelling space, and monthly income), lifestyle habits (i.e. smoking, alcohol use, dietary, tea consumption, outdoor activities, and sleep quality), and health conditions (e.g. history of chronic conditions such as stroke, and heart disease). A total of seven health conditions were included in the analysis, including obesity, hypertension, hyperlipidemia, diabetes, cataract, history of heart disease, and history of stroke. We defined obesity as body mass index of 30 kg/m^2^ or more; hypertension as systolic/diastolic blood pressure of 140/90 mmHg or more, or use of anti-hypertensive medications; hyperlipidemia as a total cholesterol level of 6.2 mmol/l or more or use of lipid-lowering drugs; diabetes as fasting blood-glucose of 7.0 mmol/l or more, or use of diabetic medications; history of heart disease and stroke as self-report of physician diagnosis. Age-related cataract was graded clinically based on the Lens Opacities Classification System (LOCS) III^31^.

### Statistical analysis

The characteristics of participants with and without cognitive dysfunction were compared using the chi-square test for categorical variables and the analysis of variance for continuous variables. Cognitive dysfunction was defined as AMT score no more than 7^21^.

To assess the association between cognitive dysfunction and older people’s HRQOL, a series of multivariate linear regression models controlling for covariates were used for both EQ-5D index and VAS scores. A binary variable was generated to indicate cognitive function status (normal cognitive function vs. cognitive dysfunction) in the models. The first model (model 1) was adjusted for socio-demographic characteristics only: age, gender, education level (no formal education vs. formal education), living with a spouse (with vs. without), working status (working vs. retired), dwelling space (≤60 m^2^ vs. 61–120 m^2^ vs. >120 m^2^), monthly income (≤1000 CNY vs. 1001–3000 CNY vs. >3000CNY). The second model (model 2) was further adjusted for lifestyle parameters: smoking (never vs. former vs. current), alcohol consumption (never vs. former vs. current), dietary (normal vs. vegetarian), habitual tea consumption (non-habitual vs. habitual), outdoor activities (with vs. without), and sleep quality (not good, normal, and good). The third model (model 3) included all covariates in model 2 plus presence or absence of health conditions: obesity, hypertension, hyperlipidemia, diabetes, cataract, history of heart disease, and history of stroke. Since the distributions of the EQ-5D index and VAS scores were skewed, the robust standard error estimator was used in the models.

We also created a categorical variable corresponding to severity of cognitive dysfunction (normal cognitive function, moderate and severe cognitive dysfunction). The impacts of cognitive dysfunction severities on the EQ-5D index and VAS scores were analyzed using repeated multivariate linear models described above.

In addition, multivariate logistic regression models were used to explore the association between the presence of EQ-5D health problems and cognitive dysfunction. In the analysis, a binary variable (with problems or without problems) was generated to specify the existence of any health problems in each of the five EQ-5D dimensions, and was analyzed in separate logistic models. All covariates in model 3 were included in the logistic models.

## Results

Overall, the study population had good health, indicated by low prevalence of incorrect responses in most of AMT items (Table 1), and of reporting EQ-5D problems in all dimensions (Table 2). As a result, the mean AMT and EQ-5D index scores were 8.55 and 0.95, respectively. Among the 5,557 participants, 1,238 (22.3%) participants had an AMT score less than 8 and thus were regarded with cognitive dysfunction. The proportions of severe and moderate cognitive dysfunction were 5.4% (n = 67) and 94.6% (n = 1,171) in participants with cognitive dysfunction.

Study participants’ characteristics according to cognitive function are shown in Table 3. The mean EQ-5D index and VAS scores for participants with cognitive dysfunction (0.936 and 75.5) were significantly lower than those for participants with normal cognitive function (0.959 and 80.3, p < 0.0001 for both). Similarly, the prevalence of any problems in EQ-5D dimensions were significantly higher for participants with cognitive dysfunction (p < 0.001 for all). The mean age, proportions of female, no formal education, retirement, and the prevalence of hypertension, hyperlipidemia, cataract, history of stroke, and current or former smoker, current alcohol drinker were significantly higher among those who were with cognitive dysfunction. Conversely, an older person with normal cognitive function was more likely to have a spouse, larger dwell space, higher monthly income, higher BMI, tea drinking habits, be with outdoor activities and good sleep quality.

The impacts of cognitive dysfunction on the EQ-5D index and VAS scores controlling for covariates are displayed in Table 4. Cognitive dysfunction consistently had significantly negative impacts on EQ-5D index and VAS scores, though the degree of impacts was slightly attenuated when more covariates were adjusted. The models also revealed that the impacts increased with the severity of cognitive dysfunction. According to the final model (model 3), the coefficients for the EQ-5D index score were −0.016 (95% confidence interval [CI]:−0.024, −0.008), −0.044 (95% CI: −0.084, −0.003), and −0.015 (95% CI: −0.024, −0.006) when all older people with cognitive dysfunction, only older people with severe cognitive dysfunction, and only older people with moderate cognitive dysfunction were considered, respectively. Similarly, the corresponding coefficients for the EQ-5D VAS score were −3.4 (95% CI: −4.5, −2.4), −9.1 (95% CI: −12.3, −5.9), and −3.2 (95% CI: −4.3, −2.1).

The odds ratios (OR) of cognitive dysfunction in predicting the EQ-5D problems in mobility, pain/discomfort, and anxiety/depression are described in Table 5. Self-care and usual activities were not analyzed because of the low proportion of participants reporting such problems (0.54% for self-care and 0.83% for usual activities). After adjusting for covariates, significantly positive associations were observed between the presence of cognitive dysfunction and reporting of problems in pain/discomfort and anxiety/depression, with the ORs being 1.37 (95% CI: 1.12, 1.69) and 2.13 (95% CI: 1.41, 3.23), respectively. The relations were stronger in older person with severe cognitive dysfunction (Table 3).

## Discussion

In this cross-sectional study of community-dwelling adults aged 60 years and above, we found that cognitive dysfunction was significantly associated with worse HRQOL. Specifically, an older person with cognitive dysfunction was more likely to experience EQ-5D health problems in pain/discomfort and anxiety/depression, and rates his/her overall health less favorable. The association was more clearly evident for severe cognitive dysfunction. To our knowledge, this is the first study to assess the association of cognitive dysfunction with HRQOL in older Chinese. With the advantage of large sample size and regression techniques adjusting for various potential confounders, our study may also clarify the unclear association according to former studies^16^,^17^,^18^.

We found that the prevalence of severe cognitive dysfunction and reporting EQ-5D health problems were low, which is not very surprising. Our study was based on a noninstitutionalized older population whose health status is better than those living in institutions such as nursing home and hospital. Also, our results are consistent with the results of previous community-based studies assessing prevalence of cognitive dysfunction^32^,^33^,^34^ or measuring HRQOL using the EQ-5D^28^,^29^ in China.

The findings that the EQ-5D and VAS scores were significant lower in older adults with cognitive dysfunction, and decreased with the severity are in consistence with the findings from a study in Sweden^16^. On the other hand, the detected EQ-5D dimensions in which participants with cognitive dysfunction are more likely to tolerate problems are not totally consistent. We found that the EQ-5D problems in all dimensions were more prevalent for cognitive impaired participants; while the study in Sweden failed to detect such the phenomena in pain/discomfort dimension, which may be due to the limited sample size of the study (n = 373).

The larger impact of severe cognitive dysfunction on HRQOL than moderate cognitive dysfunction was also similar with the findings from a previous study comparing HRQOL between older people with dementia, mild cognitive impairment, and controls in Belgium^17^. Nevertheless, the study observed no significant differences in HRQOL between mild cognitive impairment and control groups, which might be due to two reasons. First, HRQOL in that study was assessed using a disease-specific instrument Alzheimer’s Disease Related Quality of Life focusing on specific aspects related to disease; while participants with mild cognitive impairment may not have experienced the disease symptoms or progress. Second, the small size (n = 36 in mild cognitive impairment group) may have limited statistical power to detect significant associations.

The negative association between cognitive dysfunction and EQ-5D index score could be explained by the higher probability in reporting problems in the two EQ-5D dimensions (pain/discomfort, and anxiety/depression) in the older adults with cognitive dysfunction in China. Prior epidemiologic studies also revealed similar findings with respect to anxiety/depression in old people^9^,^35^ that people’s awareness of cognitive decline or impairment may cause their upsets as a psychological reaction due to the loss of cognitive functioning. A study in surgical patients also found that the patients with cognitive deficits have a greater risk of chronic pain after surgery^8^. Overlaps have been observed between brain regions involved cognition and pain modulation, hence, cognitive dysfunction may play a role in the development of chronic pain^8^. On the other hand, previous studies in nursing home populations have shown that increased severity of cognitive dysfunction was associated with decreased self-reported pain^36^,^37^. It should be noted that those studies concerned older adults living in institutions, while all participants in our study lived in their home yet. Indeed, a study based on older adults who mostly lived in their home found that participants with impaired cognitive function were more likely to report disabling back pain than those with normal cognitive function^38^. While the difference in place of residence may be a reason, the study also reported that the adjustment of residence place had little effect on the results. Hence, future studies should further explore this issue by collecting more information such as medication for pain relief used, and the non-drug pain-management strategies.

We did not detect cognitive dysfunction as an independent factor of mobility problems, though the prevalence of mobility problems was higher in participants with cognitive dysfunction than in their counterparts. Previous cross-sectional and longitudinal studies^6^,^39^ have shown cognitive dysfunction is associated with mobility function in elders. The inconsistency may be due to a few of participants were with mobility problems (4.67% and 2.14% for participants with and without cognitive dysfunction, respectively) in our study, which might have led to the failure to detect a statistically significant association.

Our findings could have important implications for policy makers who may have believed that cognitive dysfunction has little influence on HRQOL in older people as they may often have decreased awareness of their cognitive dysfunction and behavior change. Hence, efforts and resources in healthcare should be spent more to maintain the cognitive functioning or relieve the symptoms of cognitive dysfunction in older people to keep their HRQOL as relatively favorable.

The study has several limitations which should be acknowledged. First, we could not conclude a causal relationship between cognitive dysfunction and HRQOL due to the nature of cross-sectional design. On the other hand, we controlled for a variety of factors, and the findings were robust to the adjustments. Second, our results may not be generalizable to older adults living in institutions such as nursing home or hospital. Third, compared to using comprehensive neuropsychological test battery, using the AMT only should have a lower sensitivity and specificity. Nevertheless, the prevalence of cognitive dysfunction in our study (22.3%) is similar with the prevalence detected using the batteries from previous studies in China (ranging from 15.7% to 23.3%)^32^,^33^,^34^. Fourth, the prevalence of EQ-5D problems self-care and usual activities were low in our study. We therefore were unable to deduce any associations of them with cognitive dysfunction. Previous studies in both general and patient populations in China also reported similar findings^28^,^29^,^30^,^40^. For example, nobody reported having self-care problems in a sample of chronic prostatitis patients^30^. This may be partially due to the EQ-5D is insensitive to detect small to moderate difference in health status^41^.

## Conclusion

In conclusion, cognitive dysfunction, particularly severe dysfunction, is associated with worse health-related quality-of-life in older adults.

## Additional Information

**How to cite this article**: Pan, C.-W. *et al*. Cognitive dysfunction and health-related quality of life among older Chinese. *Sci. Rep*. **5**, 17301; doi: 10.1038/srep17301 (2015).

## Figures and Tables

**Table 1:** Responses on the AMT items and mean AMT score.

	Score 0 (%)	Score 1 (%)
AMT1 (age)	0.5	99.5
AMT2 (time of day)	10.0	90.0
AMT3 (year)	9.7	90.3
AMT4 (place)	1.2	98.8
AMT5 (recognition of people)	0.7	99.3
AMT6 (date of birth)	25.5	74.5
AMT7 (national day)	17.6	82.4
AMT8 (president)	27.3	72.7
AMT9 (counting backward from 20 to 1)	12.4	87.6
AMT10 (recall of an address)	40.4	59.6
Mean AMT score (SD)	8.55 (1.73)	

AMT: Abbreviated Mental Test; SD: standard deviation.

**Table 2:** Responses on the EQ-5D dimensions and mean EQ-5D index and VAS scores.

EQ-5D dimension	No problem (%)	Moderate problems (%)	Severe problems (%)
Mobility	97.52	2.45	0.04
Self-care	99.46	0.45	0.09
Usual activities	99.17	0.77	0.05
Pain/discomfort	72.85	26.51	0.65
Anxiety/depression	95.11	4.53	0.36
Mean EQ-5D index score (SD)		0.954 (0.081)	
Mean EQ-5D VAS score (SD)		79.2 (11.8)	

EQ-5D: European Quality of Life-5 Dimensions; VAS: visual analogue scale; SD: standard deviation.

**Table 3:** Characteristics of study participants.

Characteristics	Normal cognitive function (n = 4,318)	Cognitive dysfunction (n = 1,238)	p-value
Age (years) (SD)	67.1 ± 6.0	71.6 ± 7.6	<0.001
Female (%)	44.4	77.0	<0.001
No formal education (%)	39.8	80.2	<0.001
Living with a spouse (%)	86.6	69.0	<0.001
Retired (%)	63.0	79.2	<0.001
Dwelling space (%)			
≤60 m^2^	13.1	20.2	
61–120 m^2^	25.5	21.9	<0.001
>120 m^2^	61.4	58.0	
Monthly income (%)			
≤1000 CNY	52.5	78.1	
1001–3000 CNY	38.7	20.4	<0.001
>3000 CNY	8.8	1.5	
BMI (SD)	23.4 ± 3.3	22.7 ± 3.3	<0.001
Hypertension (%)	73.7	78.8	0.003
Hyperlipidemia (%)	5.9	7.7	0.026
Diabetes (%)	12.1	11.2	0.375
Cataract (%)	53.4	63.0	<0.001
History of heart disease (%)	7.9	8.8	0.315
History of stroke (%)	1.6	3.0	0.001
Current or former smoker (%)	30.0	11.6	<0.001
Current alcohol drinker (%)	26.6	9.0	<0.001
Tea drinking habits (%)	39.7	17.7	<0.001
Dietary			0.015
Normal	98.5	97.4	
Vegetarian	1.6	2.6	
Without outdoor activities (%)	56.3	61.6	0.001
Sleep quality			
Not good	8.7	13.1	
Normal	12.4	15.3	<0.001
Good	78.9	71.6	
Mean EQ-5D score (SD)	0.959 ± 0.073	0.936 ± 0.101	<0.001
Mean VAS score (SD)	80.3 ± 11.5	75.5 ± 11.9	<0.001
EQ-5D problems (%)			
Mobility	2.14	4.67	<0.001
Self-care	0.35	1.73	<0.001
Usual activities	0.6	2.27	<0.001
Pain/discomfort	24.88	38.36	<0.001
Anxiety/depression	4.01	8.06	<0.001

EQ-5D: European Quality of Life-5 Dimensions; VAS: visual analogue scale; SD: standard deviation; BMI: body mass index; CNY: Chinese Yuan.

**Table 4:** Impact of cognitive dysfunction on EQ-5D index and VAS scores in multivariate regression analysis.

	Model 1		Adjusted R^2^	Model 2		Adjusted R^2^	Model 3		Adjusted R^2^
	Coefficient (95% CI)	Standardized Coefficients		Coefficient (95% CI)	Standardized Coefficients		Coefficient (95% CI)	Standardized Coefficients	
**EQ-5D index score**									
All cognitive dysfunction (n = 1,238)	−0.017 (−0.026, −0.009)	−0.088	0.145	−0.016 (−0.025, −0.008)	−0.083	0.196	−0.016 (−0.024, −0.008)	−0.082	0.235
Severe cognitive dysfunction (n = 67)	−0.051(−0.095, −0.007)	−0.062	0.146	−0.051 (−0.094, −0.007)	−0.061	0.197	−0.043 (−0.084, −0.003)	−0.052	0.236
Moderate cognitive dysfunction (n = 1,171)	−0.016 (−0.025, −0.007)	−0.079		−0.015 (−0.024, −0.006)	−0.074		−0.015 (−0.024, −0.006)	−0.075	
**EQ-5D VAS score**									
All cognitive dysfunction	−3.6 (−4.7,−2.5)	−0.121	0.171	−3.5 (−4.5, −2.3)	−0.116	0.197	−3.4 (−4.5,−2.4)	−0.117	0.263
Severe cognitive dysfunction	−10.6 (−14.1, −7.1)	−0.086	0.175	−10.2 (−13.8, −6.7)	−0.083	0.200	−9.1 (−12.3, −5.9)	−0.074	0.265
Moderate cognitive dysfunction	−3.3 (−4.4, −2.2)	−0.109		−3.1 (−4.2, −2.0)	−0.105		−3.2 (−4.3, −2.1)	−0.107	

EQ-5D: European Quality of Life-5 Dimensions; VAS: visual analogue scale; CI: confidence interval.

Model 1: adjusted for age, gender, education level, marriage status, working, dwelling space, and monthly income.

Model 2: covariates in model 1 plus lifestyle habits: smoking, alcohol consumption, dietary, outdoor activities, and sleep quality.

Model 3: covariates in model 2 plus health conditions: obesity, hypertension, hyperlipidemia, diabetes, cataract, history of heart disease, history of stroke, and cognitive impairment.

**Table 5:** Odds ratios (95% confidence interval) of cognitive dysfunction in predicting EQ-5D health problems.

	Mobility	Pain/discomfort	Anxiety/depression
All cognitive dysfunction* (n = 1,238)	0.92(0.53, 1.56)	**1.37** (1.12, 1.69)	**2.13** (1.41, 3.23)
Severe cognitive dysfunction* (n = 67)	1.08 (0.29, 4.01)	**2.78** (1.33, 5.88)	2.44 (0.68, 10.09)
Moderate cognitive dysfunction* (n = 1,1171)	0.90 (0.52,1.56)	**1.33** (1.08, 1.64)	**2.08** (1.39, 3.23)

EQ-5D: European Quality of Life-5 Dimensions.

^*^Model 3 in Table 2 was used for adjustment; boldness: *P* < 0.05.
